# Practical Notes and Formulæ

**Published:** 1883-01-20

**Authors:** 


### PRACTICAL NOTES AND FORMULÆ.

**Treatment of Malarial Fever.**—Dr. W. L. Bell, in Medical and Surgical Reporter, says:

Having lived for years on the banks of the Mississippi river, where intermittent and remittent fevers are the most constant maladies the physician has to combat, I will give my treatment in the chronic forms of this disease, in connection with enlargement of the spleen; and where it has been persistently adhered to for five or six weeks, I cannot record a single failure.

℞ Sulphite quinine.............................. ʒ j,<br>

Crys. iodine.................................... gr. xv,<br>

Ipecac pulv..................................... gr. xx.

Triturate the iodine; add the ipecac and quinine, triturate the combination well; divide into forty pills.

Sig. One pill half hour before each meal.

I give the following prescription in connection with the above, to relieve the visceral obstruction and engorgment:

℞ Fluid ext. mandrake.........................}<br>

Fluid ext. leptandrin.......................} aa ʒ j,<br>

Fluid ext. stillingia.........................}<br>

Whisky...................................... ℥ ij.

M. Sig. Teaspoonful after each meal.

It will be necessary to check the paroxysm with an anti-periodic before commencing the above treatment. In very obstinate cases, where the disease is of long standing and the spleen is very much enlarged and indurated, an application of the comp. iodine ointment every other day will facilitate the reduction of this organ.

**Dr. Gross' Neuralgic Pills.**

℞ Dextro-quiniæ.............................. ʒ ij,<br>

Morphiæ sulphatis........................... grs. iij<br>

Strychniæ sulphatis......................... grs. ij,<br>

Acid arseniosi............................... grs. iij,<br>

Ext. aconiti.................................. grs. xxx.

M. et. div. id pil. No. lx.

IMPROVED—DR. GROSS' NEURALGIC PILL—WITHOUT MORPHIA.

℞ Dextro-quiniæ.............................. ʒ ij,<br>

Strychniæ sulphatis......................... grs. ij,<br>

Acid arseniosi............................... grs. iij,<br>

Ext. aconiti.................................. grs. xxx.

M. et div. in pil. No. lx.—*Monthly Review.*

**Hypodermic Use of Quinine—**

℞ Quinia bisulph................................grs. j,
Carbol. acid................................gtt. v,
Sulph. acid................................gtt. ii,
Aqua pura................................℥ i.

M. One hypodermic syringeful equals from 3 to 4 grs. of the sulphate given "viam natura."

During the present summer season I have been battling with pernicious fevers in a variety of forms, both intermittent and remittent. In the intermittent it has proven most efficacious. When the attack is of an algidus form, hemorrhagic, or when the congestion is located in the stomach and bowels, adding one-fourth gr. morph. with the first administration in adults, I have been fully satisfied no ulcer is produced, and there is but little inconvenience felt, though there may be some soreness for a few days at the point of injection.—*Miss. Valley Med. Monthly.*

**Emmenagogues.**—Dr. Booth, in Therapeutic Gazette, says: "In answer to Dr. Charles H. Miller's query, "What is wrong with our emmanagogues?" permit me to say the trouble lies, judging from his article, in that he prescribes too empirically, not giving proper consideration either to agents or quantities indicated in the cases being treated. Understand, I do not say he is given over to empiricism in these cases, but that such an inference may readily be drawn from his article, *vide* the Therapeutical Gazette, Sept., 1882, p. 334. The following will be found as reliable and safe as any formula we have ever known or tried:

℞ Fl. ext. polygonum punc........................ ℥ ij

Ol. sabine................................} aa ʒ ss.

Ol. rue................................}

Mix thoroughly. Sig. ʒ ss three or four times a day.

Or, when indicated, the following emulsion of savin:

℞ Ol. savin, fl. ext................................ʒ j

Spts. nitrous ether................................ʒ iij

Mucilage of acacia................................℥ j

Water, ad................................℥ vj.

M. Sig. Teaspoonful every two hours.

**Nervine and Anti-Spasmodic—**

℞ Potassii bromidi................................gr. x
Tinct. conii................................gtt. xxx
Tinct. val. ammoniæ................................gtt. xx
Aquæ camph................................℥ j

M. A favorite prescription in the Hospital of Chest Diseases, London. It is useful in epilepsy, dysmenorrhœa, chorea, hysteria and the like.—*Medical Summary.*

**Boracic Acid Cotton.**—

| | |
|---|---|
| Purified cotton wool | q. s., |
| Boracic acid | 10 parts, |
| Water | 90 parts. |

Dissolve the boracic acid in the water at a temperature of 60° C. (40° F.). Saturate the purified cotton wool with this solution, press it, dry it, and preserve it in corked bottles having a very wide mouth.—*Drug Cir.*

**Salicylic Acid Cotton.**—

| | |
|---|---|
| Purified cotton wool | 100 parts, |
| Salicylic acid | 10 parts, |
| Strong spirit | 100 parts, |
| Glycerine | 1 part. |

Dissolve the salicylic acid in the alcohol, add the glycerine to this solution, saturate the cotton wool with the liquid. Press out the superfluous liquid and dry, etc., as above.—*Ibid.*

**Iodoform in Chronic Pulmonary Affections.**—In phthisis, even at an advanced period of the disease with the presence of cavities, iodoform has given the author excellent results. In each case it diminished expectoration, and exercised a favorable influence upon the febrile manifestations. "Iodoform," he says, "diminishes the fever and affects the expectoration, which it not only diminishes in quantity but alters in character, preventing the putrefaction of its albuminoid elements. I am also convinced that the contents of the cavities in the lung exercise a powerful influence upon the production of hectic fever." In recommending iodoform in pulmonary phthisis, the author does not assert it to be a specific, but he claims that it arrests the march of this cruel malady and prolongs the life of the sufferer.

He also holds that in cases where caseous pneumonia is commencing, iodoform administered for a time proves efficacious in arresting the progress of the disease. With many individuals affected with chronic bronchitis and emphysema, it renders great service.

The formula which is employed is as follows:

| | |
|---|---|
| Iodoform | grs. iss. |
| Powdered lycopodium | grs. viij. |
| Ext. of gentian | q. s. |

Make into 10 pilules. Take 3 to 5 in the day.

If the dose is increased, gastric disorders supervene, and it is better to continue the above dose for a considerable time.—*Glasgow Med. Journ.—Medical News.*

